# A Study in Balance: How Microbiomes Are Changing the Shape of Environmental Health

**DOI:** 10.1289/ehp.119-a340

**Published:** 2011-08-01

**Authors:** Kellyn S. Betts

**Affiliations:** Kellyn S. Betts has written about environmental contaminants, hazards, and technology for solving environmental problems for publications including *EHP* and *Environmental Science & Technology* for more than a dozen years.


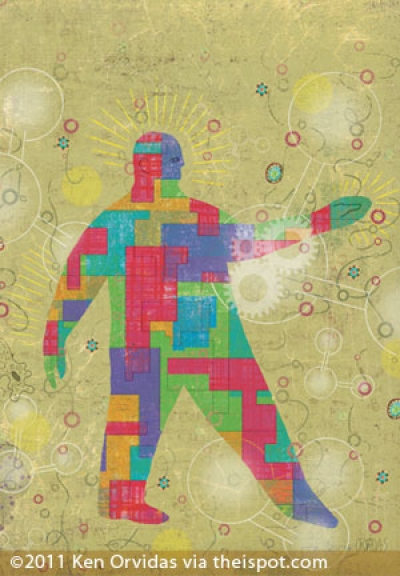
The agents we now know as bacteria have been known for centuries to play a key role in certain kinds of illnesses and ailments. But aside from infectious diseases, the communities of microbes we carry in specific parts of our bodies—our microbiomes—are a relatively new topic in human health. Now this field of study has taken an evolutionary leap forward with new research showing human microbiomes may play a far greater role in environ-mental health than ever imagined.

The excitement around this field was obvious at a National Academy of Sciences (NAS) workshop on the interplay of the microbiomes, environ-mental agents, and human health held 27–28 April 2011,^1^ where talks by researchers working in this area inspired numerous “eureka!” moments. New findings about the ways in which human microbiomes transform arsenic and mercury—two of our most prevalent and well-defined external human health hazards—suggest the role of commensal bacteria may equal or exceed that of genetic polymorphisms that regulate metal transformations within the body, says Ellen Silbergeld, a professor of environmental health sciences at the Johns Hopkins University Bloomberg School of Public Health.

The implications of these new insights are staggering. Environmental health scientists may need to expand the toxicokinetics of metals and other environmental agents, as well as associated biomarkers, to include the microbial component. “This is a huge thing that has never been thought of before in environmental health sciences,” Silbergeld told workshop attendees.

Emerging findings also demand a re-examination of what it means to be exposed to environmental agents, Silbergeld says. To a toxicologist, she explains, a contaminant is only “in the body” once it has crossed from the external environment into circulating blood, or a cell, or an organ. But new findings suggest biologically relevant transformations may take place prior to absorption, when contaminants interact with the microbiome in the mouth, intestines, or other tissues. Because of the metabolic processes mediated by microbiomes, a great deal of what toxicologists attribute to human metabolism—such as methylation of arsenic—may actually take place at least in part before contaminants cross into the internal environment of our bodies.

## Our Microbiomes: Works in Progress

All body sites—including the mouth, hair, nose, ears, vagina, lungs, and skin—have their own unique microbiomes. A person’s microbiomes are at least partly transferred at birth from the mother; scientists have documented differences in the microbiomes of infants born vaginally and by caesarean section.^2^ Microbiomes go through several transitions during the first years of life, then remain relatively constant throughout much of a person’s life until about age 65.^3^

In adulthood the composition of microbiomes is influenced not only by host genetics but also by the environment, diet, and other factors.^4^^,^^5^^,^^6^^,^^7^ If the composition of a microbiome changes, the range of services it provides its human host also may shift. Lita Proctor, coordinator for the National Institutes of Health’s Human Microbiome Project,^8^ says the key bacterial organ is the intestinal microbiome; variability in this microbiome may be an important source of individual variability in human health and disease.

Margaret McFall-Ngai, a comparative animal biologist at the University of Wisconsin–Madison, says recent evidence from evolutionary biology suggests the evolution of many human genes was likely driven by interaction with the microbiome.^33^ Tom Van de Wiele of Ghent University, Belgium, adds, “Many of these genes were ‘invented’ in a time when the first higher organisms developed and were forced to interact with the omnipresent microorganisms.”

In fact, Jeremy Nicholson of Imperial College London recently published a landmark article documenting that the intestinal microbiome played a key role in promoting the expression of cytochrome P450 enzymes, which break down xenobiotic substances such as toxic chemicals.^9^ Nicholson’s team showed that when “germ-free” mice born and raised in sterile environments were colonized with intestinal microbes, their expression of cytochrome P450s increased. In addition to indicating the intestinal microbiome enhances the host’s metabolic capacity for processing nutrients and drugs, the authors speculate “microbiota manipulation” may someday help improve drug delivery in personalized health care.

**Figure:**
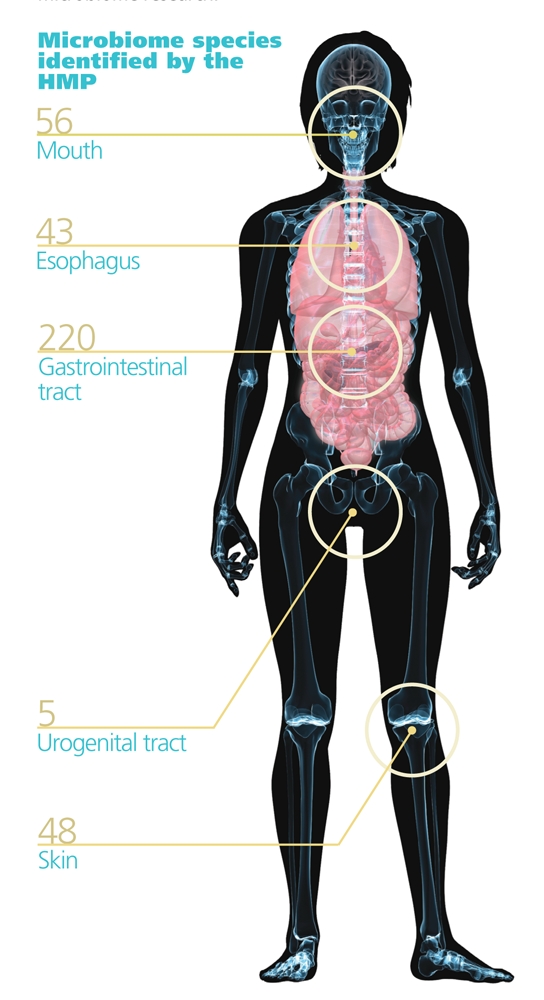
Collections of communities Each person is host to some 100 trillion microbes, predominantly bacteria. These microbes live throughout the body in communities called microbiomes. The Human Microbiome Project (HMP) of the National Institutes of Health has set four goals to better understand the role of microbiomes in human health: 1) determine whether individuals share a core human microbiome, 2) understand whether changes in microbiomes correlate with changes in health, 3) develop technological and bioinformatic tools to support these goals, and 4) address the ethical, legal and social implications raised by human microbiome research. Source: Shutterstock.com

The human intestinal microbiome appears to fall into one of three distinct enterotypes, or bacterial profiles, first defined in 2011 by the European Commission’s Metagenomics of the Human Intestinal Tract consortium.^10^ Analysis of 39 intestinal microbiome metagenomes of adults from four European countries, the United States, and Japan indicates individuals have one of these three enterotypes, which are characterized by higher proportions of *Bacteroides*, *Prevotella*, or *Ruminococcus* species.^11^ The enterotypes cross international and continental borders, as well as race, ethnicity, sex, and age. It is too soon to know how the three enterotypes differ in their ability to process environmental agents.

Scientists have long known that human intestinal bacteria synthesize important substances such as vitamin K. New evidence indicates our microbiomes are, in fact, “chemical factories” that produce a complex set of molecules, including druglike compounds and peptides that act as neurotransmitters, says Michael Fischbach, an assistant professor at the University of California, San Francisco, School of Pharmacy. Among other things, Fischbach is studying whether some of the compounds produced by the intestinal microbiome bind the aryl hydrocarbon receptor. This is potentially important because this receptor’s activation can lead to changes in gene expression that adversely affect many human cellular processes.

Other conversions performed by gut microbes can be beneficial, such as metabolization of phytoestrogen precursors from a wide variety of plants into more biolog-ically active components associated with certain health benefits. For example, isoxanthohumol is a phytoestrogen precursor occurring in hops that microbes can convert into 8-prenylnaringenin, which has known anti-inflammatory and cardio-protective properties.^12^ Recent work has documented variability in the ability of individuals to convert isoxanthohumol into 8-prenylnaringenin, a phenomenon Tom Van de Wiele, an assistant professor in the Laboratory of Microbial Ecology and Technology at Ghent University, Belgium, says may be due in part to differences in the composition of their intestinal microbiomes.^13^

One family of microbes may biotransform contaminants into a new form, whereas another group may transform that new form back into its previous form or into something completely new with completely different biological activity, according to Van de Wiele. For example, he points out the polycyclic aromatic hydrocarbons (PAHs) naphthalene, phenanthrene, pyrene, and benzo[*a*]pyrene do not typically exhibit estrogenic properties in the body. However, he conducted experiments that showed incubating these four compounds, particularly the latter two, with bacteria from the human colon resulted in the formation of compounds with estrogen-like activity.^14^ “The number of chemical conversions [in bacteria] is tremendously large. In fact, the metabolic potency is thought to exceed even that of the liver,” Van de Wiele says.

The implications of all this are dramatic when one considers how little geography matters to bacteria. “People who haven’t worked with bacteria that much tend to under-estimate what they can do,” Silbergeld says. In 2007 researchers at the U.S. Geological Survey published research documenting both the global movement of dust across continents and oceans^15^ and the fact that bacteria can hitch transcontinental rides on these dirt particles.^16^ Microbes can also just as easily travel by plane, as documented in the case of antibiotic-resistant bacteria that traveled from hospitals in India to the United Kingdom.^17^

## Microbiomes and Metals

Scientists have known for decades that certain bacteria in the environment alter metal compounds in ways that make the metals more bioaccessible to humans. The evidence that certain microbes in the human gut also can transform metals has been steadily accumulating, although only recently have environ-mental health scientists begun to grasp the implications.

One of the catalysts for this shift in thinking was the 2010 publication of a study in which Van de Wiele and his team used a “human gastrointestinal simulator” to analyze how human intestinal bacteria metabolize inorganic arsenic from contaminated soils. Different arsenic species vary widely in their toxicity, so it matters a great deal how arsenic is transformed within the body. Van de Wiele’s team showed the bacteria transformed inorganic arsenic to methylated arsenicals and thioarsenicals, including the first known observation of metabolically derived monomethylmonothioarsonic acid, whose toxicokinetic properties are unclear.^18^ When considered together with other recent research, these results suggest interindividual differences among human microbiomes may make a significant difference in the toxicity of metals and their contributions to chronic diseases associated with these metals, such as cardiovascular disease and type 2 diabetes mellitus.

Scientists agree that re-examining environ-mental exposures through the lens of the microbiome is likely to yield more insights into bacterial impacts. For instance, the ability of intestinal bacteria to demethylate methyl-mercury^19^ is important because the process could result in unexpected exposure to toxic inorganic mercury. “It is possible that [many people] may be internally exposed to inorganic mercury much more than we have ever calculated because of demethylation of the mercury we take in through fish consumption,” Silbergeld says.

**Figure:**
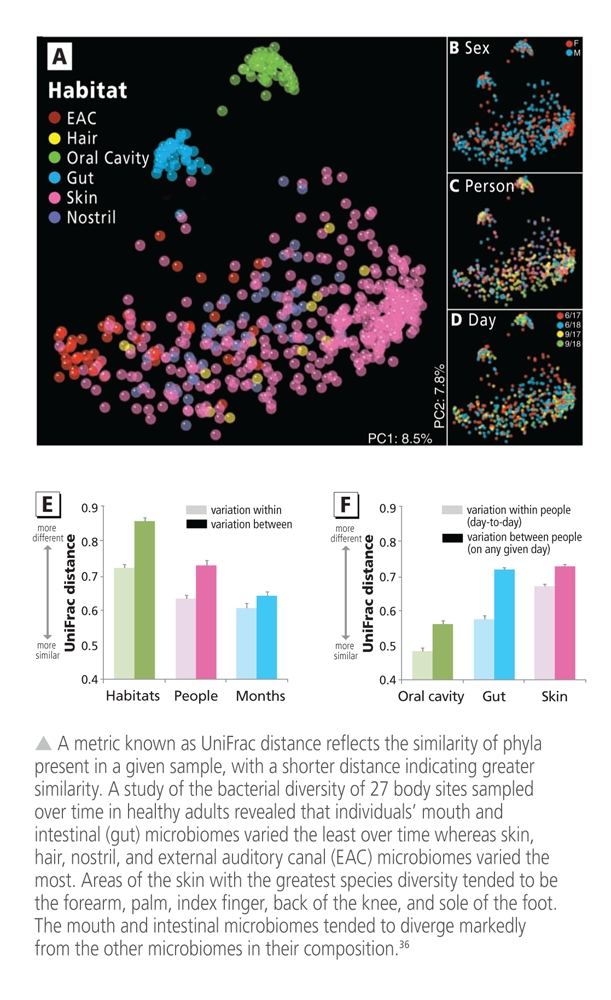
It’s personal Microbiome composition can vary widely among individuals and between different sites on a person’s body. In adulthood, the composition of any given microbiome is influenced not only by host genetics but also by the environment, diet, and other factors. The intestinal microbiome is thought to be a key player in health. Human intestinal microbiomes appear to have one of three distinct enterotypes, or bacterial profiles, characterized by prominence of a certain genus.^11^ Our microbiomes play key roles in immunity and digestion. New data suggest they also mediate metabolic processes such as methylation of potentially toxic metals. Interindividual differences in the composition of our microbiomes may be an important reason why people vary in their response to environmental stressors. Source: adapted from Costello et al. (2009)

**Figure:**
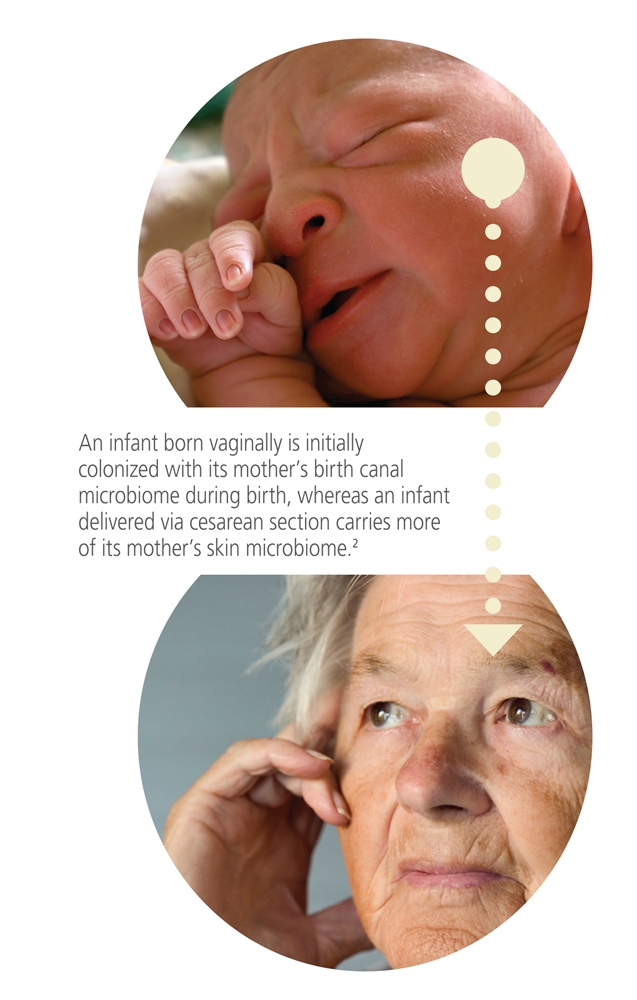
Lifetime achievement Once established, microbiomes remain relatively constant throughout a person’s life until about age 65. Source: istockphoto.com

Many people worldwide are already regularly exposed to inorganic mercury during gold-mining operations.^20^ A major percentage of Americans also may be exposed to high levels of the element via silver amalgam dental fillings in their teeth, according to an analysis by Mark Richardson, a risk assessment specialist at consulting firm SNC Lavalin.^21^^,^^22^ On the basis of an analysis he presented at a 14–15 December 2010 meeting of the U.S. Food and Drug Administration Dental Products Panel, Richardson estimated that one-quarter to one-third of Americans may be regularly exposed to concentrations of inorganic mercury from amalgam fillings that exceed recommended exposure limits set by the U.S. Environmental Protection Agency (EPA) and the California EPA.

Thousands of studies have shown that mercury affects many metabolic processes and organ systems in humans and experimental animals. Silbergeld says research by her laboratory and others indicates inorganic mercury can also impact the mucosal immune system, for instance by increasing the production of proinflammatory cytokines and serum levels of biomarkers of immune alteration related to autoimmunity. ^23^^,^^24^^,^^25^^,^^26^ On top of that, contact between mucosal cells of the immune system and the intestinal microbiome means each will affect the other, she says. To Silbergeld, this suggests interactions between environmental contaminants and the microbiome may be bidirectional.

Gary Huffnagle, a professor in the Department of Microbiology and Immunology at the University of Michigan, is studying microbiomes of the lungs—a relatively new area of study even for microbiomes, because lungs were long thought to be a relatively sterile environment. “When we look at [the lung microbiome] in terms of its composition at a genus level, the first thing you will see is that there are some individuals that have . . . very limited diversity in their lung microbiome, and others that have quite a bit of diversity,”^34^ Huffnagle told participants at the April NAS meeting.

The implications also further strengthen the argument for including immunology and infectious disease under the umbrella of environ-mental health.^27^ Linda Birnbaum, director of the National Institute of the Environ-mental Health Sciences (NIEHS), says the institute’s research is repeatedly uncovering new interactions between the immune system and diseases she says clearly have environmental components, such as autism and breast cancer. “We know there are numerous and complex relationships between the microbiome and our immune system,” Birnbaum says.

## Metals and Antibiotic Resistance

One reason microbes are so successful on this planet is that they don’t limit their gene exchange to reproduction. Instead, Proctor says, “They promiscuously share genes across all kinds of habitats and under all kinds of conditions.” They do this by sharing snippets of DNA known as plasmids, a process known as horizontal gene transfer that has nothing to do with reproduction.^28^ “Microbes do it very, very well, and they do it a lot,” Proctor says.

Plasmid sharing is one way bacteria develop resistance not only to antibiotics but to any agent that threatens their survival, including metals. In situations where intestinal bacteria are continuously exposed to a metal such as mercury, those bacteria with the genetic machinery that enables them to tolerate the metal are more likely to survive and reproduce. Anne Summers, a microbiologist at the University of Georgia, explains, “In the high-impact environments there are more complex plasmids, but the underlying machinery for generating that complexity has been enabling bacterial evolution for eons.” She says humans have generated unprecedented environments with high concentrations of antibiotics and metals, especially mercury, inside our bodies.

Summers says bacterial exposure to metals such as mercury can contribute to antimicrobial resistance because many transferrable plasmids carry genes for multiple types resistance. In other words, in the process of developing metal resistance, a bacterium may also become resistant to an antibiotic it hasn’t even encountered. This is important because the result of our collective microbiomes’ gene transfers may not always be as good for us as they are for our microbiomes, says Les Dethlefsen, a staff scientist at Stanford University. As Silbergeld puts it: “We may exist at the pleasure of the microbes.”

**Figure:**
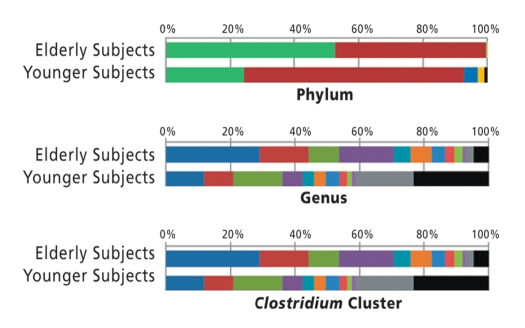
Analysis of the core intestinal microbes of elderly subjects compared with younger adults showed differences between the two age groups in proportions of phyla, genera, and clusters of highly diverse *Clostridium* species.^3^ Better understanding of the unique characteristics of the elderly intestinal microbiome may lead to recommendations for dietary interventions to improve elders’ health.

Photographs: istockphoto.com; resistance diagram: Bryson Biomedical Illustrations, Inc.; figure: adapted from Claesson et al. (2010)

Source: Bryson Biomedical Illustrations, Inc.

In studies conducted using monkeys with amalgam fillings, Summers demonstrated that exposure of the primates’ oral and intestinal microbiomes to inorganic mercury from their teeth was associated with a large increase in bacteria that were resistant both to mercury and to multiple antibiotics.^29^ Similarly, researchers at the Technical University of Denmark studying *Staphylococcus aureus* from Danish swine reported that the plasmid containing a gene for methicillin resistance also contained a gene that conferred resistance to zinc and cadmium.^30^ The authors point out that zinc oxide is widely used to prevent diarrheal diseases in livestock, and they say that both human and animal exposures to metals such as zinc and cadmium may perpetuate the spread of methicillin-resistant *S. aureus*.

Indeed, similar results are turning up in studies in human populations, such as an investigation by a team of researchers led by David Skurnik, who is affiliated with the Université Paris-Diderot and Harvard Medical School.^31^ The study involved two groups: French subjects consisting of pig farmers and bank insurance workers, and French Guyanan subjects consisting of city-dwelling French expatriates and members of the indigenous Wayampi tribe living in an isolated area. The Wayampi were heavily exposed to mercury used in artisanal gold mining, whereas all the other subjects lived in environments with low levels of mercury.

The researchers reported finding mercury-resistant *Escherichia coli* significantly more frequently in the pig farmers than in the bank insurance workers; the farmers also had higher carriage rates of antibiotic-resistant *E. coli*. In parallel, the scientists found the Wayampi participants had greater numbers of antibiotic-resistant *E. coli* than the expatriates, even though the tribe members used fewer antibiotics.^31^

Microbiomes form one component of a new concept known as the “exposome,” or the measure of all of an individual’s exposures over his or her lifetime and how those exposures affect disease risk. In the words of Stephen Rappaport and Martyn Smith, we should “consider the ‘environment’ as the body’s internal chemical environment and ‘exposures’ as the amounts of biologically active chemicals in this internal environment. Under this view, exposures are not restricted to chemicals (toxicants) entering the body from air, water, or food, for example, but also include chemicals produced by inflammation, oxidative stress, lipid peroxidation, infections, gut flora [microbiomes], and other natural processes.”^32^

## Building New Knowledge

Steve Rappaport, a professor of environmental health sciences at the University of California, Berkeley, says the environ-mental health community is now beginning to embrace a new concept that goes beyond the microbiome. This new concept is the “exposome,” which the National Institute for Occupational Safety and Health defines as the measure of all of an individual’s exposures over his or her lifetime and how those exposures affect disease risk. Rappaport says the environmental exposures that affect human health may include “all kinds of endogenous processes that have so far escaped our interest in finding the environmental causes of disease”—including the microbiome and inflammatory processes.^32^

The concept of the exposome blurs the distinction between exposure and response. “For years we talked about biomarkers, and we differentiated between biomarkers of exposure and biomarkers of response,” Rappaport says. “But when you really start drilling down into that concept of a biomarker, it can become difficult to make that differentiation.”

A recent example of this blurriness is a discovery by a group led by Stanley Hazen of the Cleveland Clinic’s Department of Cell Biology. The researchers reported identifying trimethyl-amine *N*-oxide (TMAO) as a biomarker that is highly predictive of cardio-vascular disease in Americans, and they provided evidence the intestinal microbiome plays a role in generating TMAO.^33^ The process begins with choline, an essential micronutrient, which intestinal bacterial transform catabolically to trimethylamine (TMA). TMA can be absorbed into the blood, where it is transported to the liver and metabolized to produce TMAO. But it is still unclear whether TMAO is a biomarker of the presence of choline, a particular intestinal bacterium, metabolism of TMA in the liver, factors related to arterial inflammation, or some combination of all of these, Rappaport says.

From a regulatory perspective, Rita Schoeny, senior science advisor for the EPA Office of Water, says revelations at the April NAS conference about the potential role of the microbiomes in disease risk raise many questions. For instance, EPA regulations for arsenic, and to some extent for mercury, are based on what scientists understand to be “a lovely progression of reduction and methylation with more reduction and methylation”—none of which, she says, considers microbial metabolism. Schoeny says the EPA Office of Water regulates microbes as something to be avoided, particularly in drinking water, under the assumption that the “only good bug is a nonexistent bug”; the microbiome is currently not on the agency’s radar in terms of policy making.

**Figure:**
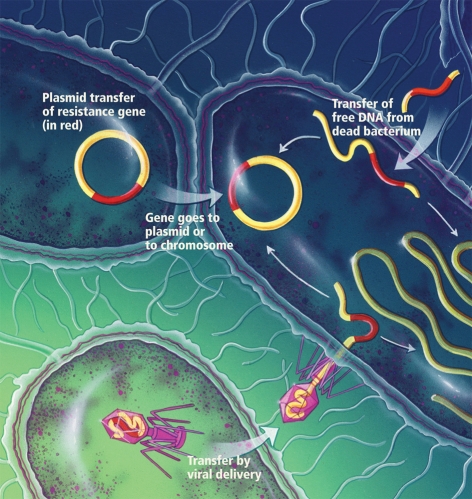
Microbiomes and resistance Commensal bacteria can develop resistance to antibiotics, metals, and other environmental stressors. One way they do this is by exchanging fragments of DNA called plasmids (they can also transfer resistance genes packaged in viruses or acquire segments of DNA released from dead cells). Plasmids can carry different genes conferring resistance to multiple agents, which means bacteria can become resistant to stressors they haven’t even encountered. Source: adapted from Claesson et al. (2010)

Vincent Cogliano, director of the EPA’s Integrated Risk Information System, told participants at the April meeting he is concerned about the absence of microbiome information in the interpretation of toxicity testing results. “Regulatory agencies are putting a lot of effort into understanding the sequence of mechanistic events that lead to disease. It looks like we have been ignoring a big number of these events that could be there,” he said. He likened the current approach to trying to write a novel without any prepositions. “You can put some coherent thoughts together,” he said, “but you cannot tell a story that is very complex or very interesting.”

How might our growing awareness of microbiomes inform public health policy? Bruce Fowler, assistant director for science in the Division of Toxicology and Environmental Medicine at the Agency for Toxic Substances and Disease Registry, says it comes down to translational risk assessment. “We recognize that there are individuals within the population who are especially sensitive to chemicals as a result of age, life stage, diet, nutrition, what have you,” Fowler said at the April meeting. “I think the microbiome just quite simply hasn’t been plugged into this, and I think it should be.”

Lisa Helbling Chadwick, the NIEHS liaison for the Human Microbiome Project, says the institute is increasing its focus on the impact of microbiomes on toxicology. “One thing we are really interested in at NIEHS is understanding how individuals respond differently to exposures, what makes one person more susceptible to adverse health outcomes from an exposure than another,” she says. “Genetics only partially explains this.” Birnbaum adds, “We recognize the enormous implications of the growing awareness of interactions between chemical exposures and the microbiome, and we have begun exploring these issues.”
